# Candidate-Probiotic Lactobacilli and Their Postbiotics as Health-Benefit Promoters

**DOI:** 10.3390/microorganisms12091910

**Published:** 2024-09-19

**Authors:** Lili Dobreva, Nikoleta Atanasova, Petar Donchev, Ekaterina Krumova, Radoslav Abrashev, Yordanka Karakirova, Ralitsa Mladenova, Vladimir Tolchkov, Nikola Ralchev, Vladislava Dishliyska, Svetla Danova

**Affiliations:** 1 The Stephan Angeloff Institute of Microbiology, Bulgarian Academy of Sciences, 26 Acad. G. Bonchev Str., 1113 Sofia, Bulgaria; ldobreva@microbio.bas.bg (L.D.); nikoletaatanasova21@gmail.com (N.A.); ekrumova@abv.bg (E.K.); ralchev.nr@gmail.com (N.R.); vladydacheva@yahoo.com (V.D.); 2 Institute of Catalysis, Bulgarian Academy of Sciences, 11 Acad. G. Bonchev Str., 1113 Sofia, Bulgaria; daniepr@ic.bas.bg (Y.K.); ralitsa@ic.bas.bg (R.M.); 3 National Center of Infectious and Parasitic Diseases, Yanko Sakuzov Blvd 26, 1504 Sofia, Bulgaria; tolchkov@ncipd.org

**Keywords:** Lactobacilli, postbiotics, metabiotics, antimicrobial, antioxidant activity, EPR spectroscopy, MTT assay

## Abstract

*Lactobacillus* species are widely recognized for their probiotic potential, focusing on their mechanisms of health benefits and protection. Here we conducted an in vitro investigation of the probiotic potential with a role in microbiome homeostasis of four strains: *Lactiplantibacillus plantarum* L6 and F53, *Ligilactobacillus salivarius* 1, and *Lactobacillus helveticus* 611. A broad spectrum of antibacterial and antifungal activity was determined. The strain-specific inhibition of *Staphylococcus aureus*, *Streptococcus mutans*, *Escherichia coli*, *Pseudomonas aeruginosa*, and saprophytic/toxigenic fungi makes them promising as protective cultures. DPPH (2,2-diphenyl-1-picrylhydrazyl) and ABTS (2,2′-azino-bis-(3-ethylbenzothiazoline-6-sulfonic) acid) measurements showed that tested samples had strain-specific capacity for scavenging of radicals. The molecular base for the antioxidant potential of two lyophilized forms of active strains was investigated by electron paramagnetic resonance spectroscopy. The MTT (3-(4,5-dimethylthiazol-2-yl)-2,5-diphenyltetrazolium bromide) assay, with fractions of the most active postbiotics obtained by SEC-FPLC (fast protein liquid chromatography) analysis, showed a wide variety of effects on the growth of a K562 myeloid leukemia cell line. The IC_50_ (half-maximal inhibitory concentration) of *L. salivarius* 1 was determined to be 46.15 mg/mL. The proven in vitro functionality of the selected lactobacilli make them suitable for development of target probiotics with specific beneficial effects expected in vivo. Further investigations on produced postbiotics and safety have to be completed before they can be considered as scientifically proven probiotic strains.

## 1. Introduction

Lactic acid bacteria (LAB) are among the recognized symbiont partners of humans and animals and represent an important component of the microbiome. The gut microbiota plays a dual role in the host’s health protection, producing various metabolites (metabiotics) [1]. The family *Lactobacillaceae*, with 33 genera and ~300 species (260 of them from the former genus *Lactobacillus*), are among the most actively studied microorganisms with probiotic potential. As probiotics (from ‘pro’, meaning ‘for’, and ‘bio’, meaning ‘life’), in the process of their viability in vivo, modifying the nutrient supply, and converting metabolites, they produce various by-products. A broad spectrum of bacteriocin-like inhibitory substances (BLIS) have been characterized, from volatile fatty acids to peptides and/or newly synthesized low- or high-molecular-weight active molecules with antimicrobial activity. These substances are excreted during the growth of LAB in different media and conditions [2], and their species- or strain-specific inhibitory activity against *Listeria monocytogenes*, *Staphylococcus aureus*, *Bacillus cereus* and members of the family *Enterobacteriaceae* (*Escherichia coli*, *Pseudomonas aeruginosa*, *Salmonella* spp., *Klebsiells* spp.) have been reported [3,4]. Beneficial effects of alcohols, diacetyl (2,3-butanedione), hydrogen peroxide, and lactones have been identified in antifungal studies [5]. In addition, reuterin, phenyllactic acid, and exopolysaccharides have also been described as major compounds with antifungal properties [6].

Together with cell lysates, bacterial cell-wall fragments, delivered after the death of good bacteria (named postbiotics), implement various barrier mechanisms for healthy gut homeostasis [7]. LAB impact host health by interacting with host cells [8]. Such host–microbe interactions are crucial for protection against environmental and opportunistic resident pathogens, providing an immunomodulation system [9].

LAB can influence several features of intestinal health, including its architecture and development, physiology, cellular features, metabolism, and immune homeostasis [10]. Through the host’s diet, they participate in essential metal ion uptake, such as manganese, zinc, and iron. New data show that changes in the levels of these physiologically essential metals are often associated with changes in microbial community composition, susceptibility to infection, and gastrointestinal disease [11]. Human physiology is regulated by abundant LAB species in the gut [12], and in cases of dysbiosis, is significantly affected by administered good bacteria. Our understanding of the beneficial roles of LAB in the prevention of the GIT (gastrointestinal tract) imbalance continues to evolve. Lactobacilli, as resident commensals, are capable of producing hormone-like metabolites, including short-chain fatty acids (SCFAs), gamma aminobutyric acid (GABA), and others, as a result of fermentation in the colon [13].

The specific diet and individual composition of the microbiota determine the nature of the metabolites produced [14]. Different active metabolites produced during fermentation are receiving increasing attention due to their beneficial effects on human health. LAB and their postbiotics have been reported for their antiviral [15], antibacterial [16], and antifungal activity [17].

However, little is known about their involvement in the prevention of the negative consequences of cellular oxidative stress. This stress results from an imbalance between the production and elimination of reactive oxygen species (ROS), which are primarily scavenged by the endogenous antioxidant defense system. Gut dysbiosis can lead to increased production of ROS, contributing to inflammation and oxidative damage. Thus, chronic low-grade inflammation occurs, which, in turn, can increase oxidative stress. Inflammatory cytokines released in response to dysbiosis can enhance ROS production, further exacerbating oxidative stress. Various approaches are sought to understand the mechanisms of the beneficial action of live bacteria and of their metabolites/postbiotics as a factor in the prevention of dysbiosis and its associated negative consequences, first in the gut. Those include malignancies, which remain the major causes of illness and death, with great clinical importance worldwide [18]. In 2018, there were 18.1 million new cases of cancer worldwide; by 2040, 29.4 million cases are predicted [19]. Leukemia accounts for about 3.1% of all cancers worldwide. It is responsible for about 3.9% of all cancer-related deaths globally [20].

Widely discussed predisposing factors of cancer include infectious agents and inflammation, toxic substances, ultraviolet radiation, generated ROS, and imbalance. ROS may promote low to moderate levels of abnormal cell proliferation and differentiation by inducing DNA mutations and oxidative damage, which accompanies inflammation and cancer development. The interplay between gut microbiota and oxidative stress may influence cancer risk. Increased inflammation and oxidative damage to DNA were debated as contributors to cancer development, particularly in the gastrointestinal tract. Available literature has questioned the role of friendly bacteria in cancer prevention, in improving the effects of anticancer therapy, and/or in reducing its toxicity and negative effects [21]. Oxidative damage plays a significant role in the pathology of many human degenerative diseases, such as autoimmune diseases, cancer, atherosclerosis, arthritis, etc. [22,23]. Evidence for the existence of the link between gut microbiota, oxidative stress, and health conditions highlights the importance of maintaining a balanced microbiota for overall health. As proposed in several in vitro studies, probiotic lactobacilli might activate anticancer mechanisms in the human gut, either by preventing the carcinogenesis processes or by directly inhibiting the proliferation of cancer cells [24,25], while certain strains have been shown to possess significant immunomodulating effects [26]. It has been shown that lactobacilli can counteract the abnormal metabolism of cancer cells through various mechanisms, inducing apoptosis [27], alteration in differentiation [28], or cell cycle [29]. Thus, characterization of LAB activity may promote use of pro- and postbiotics to prevent some adverse processes, which are a proven base for growing noncommunicable diseases.

The aim of the present study was to characterize in vitro candidate probiotic strains with functional characteristics as promoters of natural barrier mechanisms and health benefits in the human microbiome. In search of beneficial microorganisms producing active metabolites with anti-proliferative/cytotoxic effects and antioxidant activity, four strains of lactobacilli were selected from different habitats, and spectra of antagonistic activity (antibacterial, antifungal) and radical scavenging activity were evaluated. Their in vitro effect on the vitality of the K562 myoblastic leukemia cell line was characterized.

## 2. Materials and Methods

### 2.1. Microorganisms, Cell Line, Media, and Culture Conditions

Lactobacilli from the laboratory collection of The Stephan Angeloff Institute of Microbiology (SAIM) were selected for this study. They belong to the species *Lactiplantibacillus plantarum* strain L6 [30], *Ligilactobacillus salivarius* 1 and *Lactiplantibacullus plantarum* F53 [31], and the newly isolated *Lactobacillus helveticus* strain 611 (unpublished data). They were stored at −20 °C in De Man–Rogosa–Sharpe (MRS) agar, supplemented with 20% *v*/*v* glycerol. Different indicator microorganisms were used in the assessment of antimicrobial activity of *Lactobacillus* strains: (i) Gram (+): *Staphylococcus aureus* NCTC 6571 (Millipore, Sigma-Aldrich, Buchs, Switzerland), *Streptococcus mutans* DSMZ 20523, and Gram (−) bacteria: *Escherichia coli* WDCM 00013 (Millipore, Sigma–Aldrich, Buchs, Switzerland), *Pseudomonas aeruginosa* WDCM 00114 (Millipore, Sigma–Aldrich, Buchs, Switzerland); (ii) molds: *Aspergillus fumigatus 3–2*, *Fusarium oxysporum* NBIMCC 124 (Bulgaria), *Alternaria alternata* NBIMCC 110 (Bulgaria). Bacteria were stored at −20 °C in Brain Heart Infusion (BHI) broth (Difco), supplemented with glycerol (20% *v*/*v*), or in lyophilized form (pellets), according to the provider (Aquachim LTD, Sofia, Bulgaria). Prior to the tests, bacteria were pre-cultured twice in sterile BHI broth. The fungal strains were stored in MicrobankTM system at −20 °C.

The filamentous fungi were cultivated in potato dextrose agar (PDA) at 28 °C in PDA broth (HiMedia, Mumbai, India) for up to 7 days. After the fungal cultures’ sporulation, a spore suspension (10^6^ spores/mL) was prepared and stored at 4 °C until it was used in the tests.

#### Cell Line, Media, and Conditions

The K562 cell line was purchased from the Deutsche Sammlung von Mikroorganismen und Zellkulturen (DSMZ, Braunschweig, Germany). The cell line K562 is an experimental model of chronic myeloid leukemia. The cells were cultured in RPMI-1640 medium containing 0.3 g/L L-glutamine and 10% *v*/*v* fetal bovine serum (non-USA origin, Sigma F9665, Darmstadt, Germany). Before the experiment, a growth curve of K562 cells was generated to determine the optimal cell density.

### 2.2. Production of Postmetabolites (Cell-Free Supernatants, CFSs) and Postbiotics for In Vitro Tests

Prior to the experiments, each of the stock cultures was cultivated two or three times at 37 °C in laboratory MRS broth (HiMedia, Mumbai, India), pH 6.5. The cell-free supernatants (exponential 24 h and stationary phase 48 h cultures) were collected, and cells were harvested by centrifugation at 10,000× *g* for 10 min (Hermle, Wehingen, Germany). Filtered through a 0.22 µm bacterial cellulose acetate filter (MS^®^ CA Syringe Filter, Membrane Solutions, Auburn, WA, USA), CFSs were used in the evaluation of antagonistic activity, MTT assay, and radical scavenging activity tests. The CFSs were neutralized to pH 6.0 ± 0.2 with 5M NaOH.

#### 2.2.1. Preparation of EPS/EPS–Protein Crude Extract from LAB Spent Cultures

Collected CFSs from LAB culture were incubated with 96% ethanol (1:3 *v*/*v*) at −20 °C overnight. EPS were obtained by centrifugation at 17,000× *g* for 20 min (Hermle, Wehingen, Germany) at 4 °C. The dried precipitates were stored at −20 °C and resuspended in sterile dH_2_O prior to testing.

#### 2.2.2. Protein Estimation of Produced Postbiotics

A commercial HiMedia Bradford kit (HiGenoMB, HiMedia, Maharashtra, India) for protein estimation was used, according to the manufacturer’s instructions. A standard curve with BSA (Bovine serum albumin; mg/mL) was built, and the concentration is expressed in mg/mL.

#### 2.2.3. Lyophilization and Sample Preparation for Electron Paramagnetic Resonance (EPR) Analysis

The cells of two *Lactobacillus* cultures (*L.s.* 1 and *L.pl.* L6) in MRS broth were harvested by centrifugation at 10,000× *g* (Hermle, Wehingen, Germany). They were inoculated in 10% *v*/*v* sterile skimmed milk (Serva, Heidelberg, Germany), incubated at 37 °C for 6 h, and subjected to a freeze-drying protocol with cryoprotective media for 48 h. The frozen at −80 °C biomass was freeze-dried (lyophilized) in a vertical freeze dryer (BIOBASE Group, BKFD18P, Jinan, Shandong, China) under high vacuum (0.05 mbar) for 48 h according to the protocol of Schuelter et al. (2019) [32]. The lyophilized cells in powder (moisture < 2.3%) were stored in vacuum-sealed plastic bags at −20 °C prior to their use for EPR analyses. The living cells in both samples were >10^9^ CFU/mL. Two samples of tested lyophilized strains were subjected to EPR spectroscopy analyses.

### 2.3. In Vitro Antimicrobial Activity of LAB

The inhibitory potential of LAB was established for (i) cell-free supernatants (CFSs) with an acidic pH (marked aCFSs) and neutralized CFSs (nCFSs), with 5N NaOH to pH 6.0, obtained from LAB cultures in MRS broth (24–48 h, 37 °C); (ii) fermented milks (skimmed and whole milk); and (iii) postbiotics: crude exopolysaccharide (EPS) and cell lysates (exponential LAB cells ultrasonically destroyed with a Bandelin Sonopuls HD 2070 homogenizer; BANDELIN electronic GmbH & Co. KG, Berlin, Germany) for 30′/30′ at 4 °C).

#### 2.3.1. Antibacterial Activity

The antibacterial activity was determined in vitro as follows: by spectrophotometric growth inhibition monitoring using an adapted microplate method and different variants of an agar diffusion method, according to Todorov et al. (2013) [33] and Dobreva et al. (2023) [3]. Briefly, the growth of test-pathogens was monitored spectrophotometrically with a microplate reader (INNO, Seongnam-si, Republic of Korea) at λ = 600 nm for 24–48 h in the presence of 10% *v*/*v* metabiotics. The controls revealed the pathogens’ growth in the presence of 10% *v*/*v* MRS broth. Overnight cultures of test-pathogens (18 h) were standardized according to 0.5× McFarland (HiMedia, Mumbai, India). They were used as inoculum in both variants of tests as 10% *v*/*v* in 96-well microplates (Costar^®^, Corning Incorporated, Corning, NY, USA) or (1% *v*/*v*) in petri dishes with BHI agar/MacConkey agar (for *E. coli*), with a final concentration of 10^6^–10^7^ CFU/mL. Aliquots (50 µL) of tested postbiotics/metabiotics were added to wells in the agar medium with a diameter of 6 mm. After incubation for 24–48 h, the antimicrobial activity was reported by measuring non-growth zones (mm) around the well in triplicate. As negative controls, sterile, non-fermented milk and/or MRS broth were used. The activity of the tested CFS was compared with the positive controls, antibiotics (chloramphenicol (30 μg/disc, BulBio, Sofia, Bulgaria) and ampicillin (10 μg/disc, BulBio, Sofia, Bulgaria).

#### 2.3.2. Antifungal Activity

In vitro tests were carried out in modification, according to the protocol of Tropcheva et al.; (2014), using both variants: monolayer (PDA agar, Oxoid, Hampshire, UK) and two-layer agar method (PDA and MRS agar) [34]. According to the two-layer method (used for testing against *F. oxysporum*), the petri dishes were half-filled with cooled MRS (HiMedia, Mumbai, India), in which 10% *v*/*v* exponential culture of LAB was inoculated (24 h, 37 °C). The layer with exponential living LAB cultures was overlayed with PDA, and 3 µL of spore suspension of *F. oxysporum* was inoculated on top of the petri dishes.

In the monolayer method, LAB cells/CFS (10% *v*/*v*) were inoculated in PDA medium (used for tests vs. *A. alternata* and *A. fumigatus*). A freshly prepared spore suspension into PBS, pH 7.0 (10^6^ spores/mL), was used to inoculate (3 µL) onto the center-top of the petri dishes prepared for monolayer and two-layer variants (with 10% *v*/*v* CFS or LAB cultures inoculated into agar before solidification). The diameters (d) of growing colonies were measured daily in three replicates. In vitro tests were conducted until the controls, which contained 10% *v*/*v* MRS broth (HiMedia, Mumbai, India), filled the petri dishes (d = 90 mm). The molds’ growth inhibition was presented as % with the following formula:

Antifungal activity (%)=[(dControl−dSample)/dControl]×100

### 2.4. Free Radical Scavenging Ability Using DPPH and ABTS Methods

Two widely accepted methods (ABTS and DPPH) were applied to estimate the potential of LAB metabiotics to scavenge free radicals and determined by recording the change in the optical density (OD) (UV-Vis Spectrophotometer, Shimatsu, Japan) as follows: (i) at 517 nm using DPPH radical (1-diphenyl-2-picrylhydrazyl−0.2 mM, Sigma-Aldrich, St. Louis, MO, USA), according to the method of Brand-Williams et al. (1995) [35] and (ii) at 734 nm−ABTS radical scavenging assay using 2,2′-azino-bis 3-ethylbenzothiazoline-6-sulfonic acid, as described by Re et al. (1999) [36]. A positive control of ascorbic acid (0.1 mg/mL) was used. The radical scavenging activity (%) was quantified by using the following formulas:

DPPH activity (%)=(ODControl−ODSample)/ODControl×100

ABTS activity (%)=(ODControl−ODSample)/ODControl×100

### 2.5. Electron Paramagnetic Resonance Spectroscopy Analysis (EPR)

EPR measurements were done on a JEOL JES-FA 100 EPR (JEOL LTD, Tokyo, Japan) spectrometer operating at X-band frequency (ν ≈ 9.4 GHz) and 100-kHz field modulation. The spectrometer is equipped with a standard TE_011_ cylindrical resonator. The sample (20 mg) was placed in a special quartz EPR tube with an i.d. = 4 mm. The Varied Temperature Controller ES-DVT4 (JEOL Ltd., Tokyo, Japan) was used to permit the detection of EPR spectra at various temperatures. The desired temperature can be easily obtained by sending liquid nitrogen to the sample area, at temperatures controlled by the EPR spectrometer data system computer. The spectra were recorded at room temperature and at 123 K. No changes were observed in spectral resolution at lower temperatures. The EPR spectra were recorded under the following conditions: modulation frequency 100 kHz, microwave power 0.5 mW, modulation amplitude 0.2 mT, time constant 0.1 s, and sweep time 2 min. The EPR spectra of the lyophilized samples in a solid state were recorded.

### 2.6. Fast Protein Liquid Chromatography (FPLC) Analysis

The cell-free spent cultures of *Lactobacillus* cultures in MRS, were collected by two-step centrifugation: >at 9000× *g* for 5 min (Hermle, Gosheim, Germany) to avoid the presence of cells or other compounds from the growth medium, and at 16,000× *g* for 20 min following an ultrafiltration step using a Millipore system (Burlington, MA, USA), with a Durapore cassette (Burlington, MA, USA) > 1 million Da, to remove unwanted impurities. An FPLC system Bio-Rad model NGS Quest 10 Plus system with Size Exclusion Chromatography (SEC) with columns (ENrich SEC 70 and ENrich SEC 650; Bio-Rad Laboratories, Inc., Hercules, CA, USA) was used, following the process parameters: column calibration and working buffer 0.05 M potassium phosphate buffer with 0.1 M NaCl, pH 7.5. Sample-0.8 mL/run; at flow rate 1 mL/min. The columns were pre-equilibrated with 0.05 M potassium phosphate buffer (pH 7.5). All fractions were collected manually, concentrated, and analyzed for in vitro activity. A gel filtration standard (Bio-Rad, Laboratories Inc., Hercules, CA, USA, cat No 1511901) was applied for standard curves.

### 2.7. MTT Assay

K562 cells in the exponential growth phase were seeded in 96-well, flat-bottom plates, at a density of 3 × 10^5^ cells/mL. The seeded cells were incubated for 24 h in a CO_2_ incubator (ICO105med, Memmert GmbH, Büchenbach, Germany) in a humidified atmosphere (5% CO_2_) at 37 °C and then different concentrations of CFS from exponential/stationary phase were added at a volume of 4 µL/well. Following a 72 h incubation interval, 10 µL of MTT dye (3-(4,5-dimethylthiazol–2-yl)-2,5-diphenyltetrazolium bromide, HiMedia, Maharashtra, India) was added (5 mg/mL dissolved in PBS) to each well and incubated for an additional 4 h at 37 °C. At the end, to dissolve the formazan crystals, 100 µL of 2-propanol puriss. p.a. (Honeywell Riedel de Haen TM, Germany) containing 5% formic acid was added to every well, and absorption at 540 nm was measured using a microplate reader (INNO, Seongnam-si, Republic of Korea).

### 2.8. Statistical Analysis

All in vitro tests were measured in triplicate, in eight replicates for the MTT assay, and results are presented as means ± standard deviation (SD). Data were subjected to a Shapiro–Wilk normality test. According to the results of the normality test, one-way ANOVAs followed by Tukey’s post hoc test were applied to assess the statistical significance of differences among groups. Statistical analysis was performed using GraphPad Prism 8 (GraphPad Software, San Diego, CA, USA), and *p* < 0.05 was considered significant.

## 3. Results and Discussion

The present work characterizes, in vitro, four selected lactobacilli possessing functional characteristics that underline important probiotic mechanisms for maintaining healthy homeostasis in the microbiome. Our scientific hypothesis is that invasion of pathogens in vivo is usually associated with an inflammatory process, with several negative consequences. In combination, oxidative stress and formation of reactive oxygen species (ROS) and free radicals may promote dysbiosis and/or various malignant processes. Thus, abnormal division and cell growth occur in the affected organs. The probiotic activity of LAB microbiota against such unhealthy processes will be the subject of new research, aiming at the selection of strains whose functional characteristics will contribute to the natural protective barrier and health effects.

### 3.1. In Vitro Assessment of Four LAB Strains as Natural Antagonists of Different Pathogenic Bacteria and Molds

**Figure 1:**
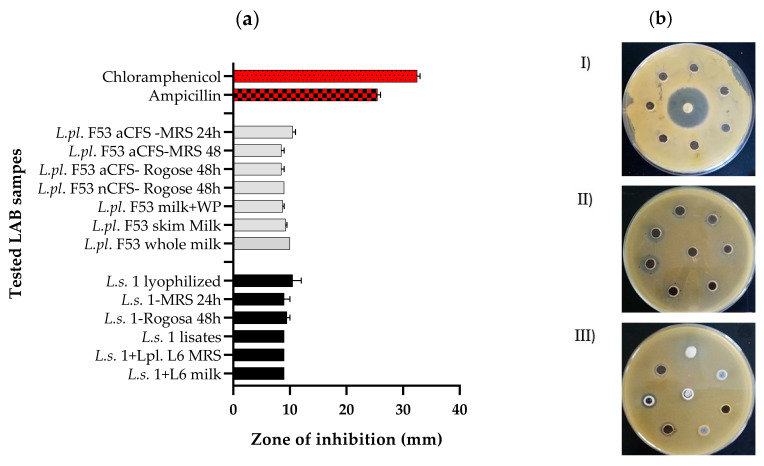
(**a**) Antagonistic activity of metabiotics of pre-selected lactobacilli against *Staphylococcus aureus* NCTC 6571, by the agar-well diffusion method. (**b**) Illustration of the inhibition zones obtained with ampicillin (10 μg/disc) as a positive control (I), tested CFS (II); and tested fermented milk, lyophilized CFS, and postbiotics (III). The antibacterial effects of the tested metabiotics (aCFS-acid, nCFS neutralized, and lysates) were expressed in mm diameter of inhibitory zones (with a well diameter of 6 mm). Two positive controls were used: the antibiotics chloramphenicol (30 μg/disc) and ampicillin (10 μg/disc) (BulBio, Sofia, Bulgaria)—the red bars. Gray bars show inhibition zones (mm) for samples derived from *L.pl. F53* and the black bars—inhibition zones (mm) for samples derived from *L.s.* 1.

**Figure 2:**
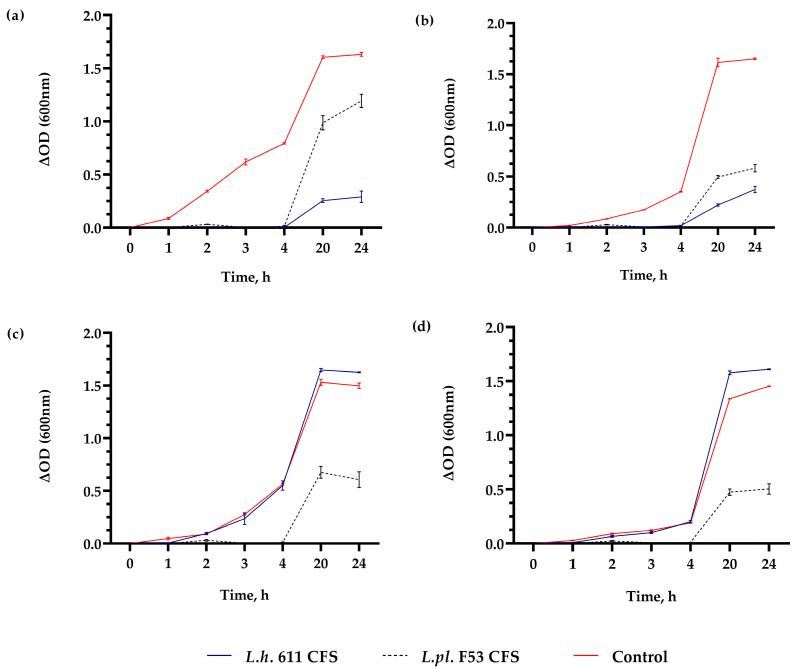
Antibacterial activity of postmetabolites of LAB cultures in MRS broth (10% *v*/*v* added to BHI broth) against (**a**) *Escherichia coli* WDCM 00013, (**b**) *Staphylococcus aureus* NCTC 6571, (**c**) *Streptococcus mutans* DSMZ 20523, and (**d**) *Pseudomonas aeruginosa* WDCM 00114. Data are presented as means ± SD of spectrophotometric measurements in triplicate (for each sample and time point). The controls indicate growth (OD 600 nm) of test pathogens in BHI broth supplemented with MRS broth 10% (*v*/*v*).

Determination of the spectrum of antimicrobial activity is an essential step in the characterization of LAB as probiotics [37]. Thus, different test cultures—*Escherichia coli* WDCM 00013, *Staphylococcus aureus* NCTC 6571, *Streptococcus mutans* DSMZ 20523, and *Pseudomonas aeruginosa* WDCM 00114, were selected as representatives of food spoilage and pathogenic microorganisms for our study. Results from in vitro assays conducted in triplicate, with the four selected pathogens, clearly demonstrate that the spectrum of antimicrobial activity is strain-specific (Figure 1 and Figure 2). Based on some preliminary results, *Lactiplantibacillus plantarum* L6 and *Ligilactobacillus salivarius* 1 were characterized as active antagonists against *Salmonella enterica* subsp. *enterica* serovar Typhimurium WDCM 00031, *Escherichia coli* HB101, and *Pseudomonas aeruginosa* PaO1 [3,38]. Moreover, they possessed a synergic anti-*Staphylococcus aureus* inhibitory effect as shown by the agar-well diffusion test (Figure 1). The results of the assessment of different postbiotics revealed a broad spectrum of activity for *L. plantarum* L6, *L. salivarius* 1, *L. plantarum* F53, and *L. helveticus* 611, which composed the group of four tested strains. Despite the significant difference with the zone generated from the commercial antibiotics chloramphenicol and ampicillin, the results showed stable inhibitory effects expressed by different metabolites/postbiotics of the tested lactobacilli. The mechanisms by which LAB inhibit the development of pathogens are diverse, with some of them being the production of inhibitory compounds, prevention of pathogen adhesion, competition for nutrients, stimulation of the host immune system by inducing cytokine production, and reduction in toxin concentration [39]. Therefore, with the use of such easy and reproducible methods, we may select new candidate-probiotic lactobacilli from different genera and species with high antagonistic potential. The obtained results with acid and neutralized CFS of *L. plantarum* F53 (Figure 1) confirm a possible BLIS, low molecular mass molecule production, or active metabolites different from volatile acids. However, further studies are needed to accurately determine BLIS production, which are upcoming.

The panel of four test-pathogens was carefully selected. The human pathogens *S. aureus* and *E. coli* are causative agents of various infections and are invasive and highly adaptable to different hosts and environmental conditions. Due to their ability to rapidly develop antibiotic resistance, they are among the main causative agents of hospital- and community-acquired infections [40]. An *S. mutans* test-pathogen was also included, due to the recently growing interest in homeostasis in the oral microbiome as a factor of human health. Another Gram (−) bacteria, *P. aeruginosa*, was added as a pathogen, which is characterized by great genetic flexibility and a variety of virulence factors capable of exhibiting antibiotic resistance to most antibiotics.

In addition to the agar-well diffusion assay, a spectrophotometric assessment of the pathogens’ growth inhibition by *L. helveticus* 611 and *L. plantarum* F53 was carried out (Figure 2). The spent CFS from *L. plantarum* F53, added to the BHI broth (10% *v*/*v*), significantly inhibited the growth of four selected test pathogens (Figure 2). In the presence of postbiotics, pathogens showed a prolonged lag phase and a retarded exponential growth phase. However, growth inhibition was not observed when CFS from *L.h.* 611 was added. With variants of the agar-diffusion method (Figure 1) and the spectrophotometric monitoring of the growth inhibition of four pathogens (Figure 2), the spectrum of activity against Gram (+) and Gram (−) bacteria was estimated. We assessed the activity of *L. salivarius* 1 and *L. plantarum* F53 in various forms (cell lysates, lyophilized form, milk, etc.) toward *S. aureus* (Figure 1), which have practical significance as well-targeting postbiotics. Considering the wide range of produced metabolites that contribute to the antimicrobial ability of *Lactobacillus* spp., such as organic acids, ethanol, diacetyl, carbon dioxide, hydrogen peroxide, and BLIS/bacteriocins [41,42], additional tests are needed to characterize which of them are synthesized by the tested lactobacilli. The production of bacteriocins/H_2_O_2_ and other inhibitory metabolites is a strain-dependent feature and requires strain-level rather than species-level screening.

In vitro assays confirmed a stable production of antimicrobial postmetabolites against *Staphylococcus aureus* in two laboratory growth media, MRS and Rogosa, from the exponential to the stationary phase of the strains *L. plantarum* F53 and *L. salivarius* 1 (Figure 1). In addition, inhibitory activity against *Lactobacillus delbrueckii bulgaricus*, *Streptococcus thermophilus* (a symbiotic starter for Bulgarian yogurts), *Lactobacillus acidophilus* ATCC 4356^T^, *Lactobacillus crispatus*, and *Lactobacillus gasseri* (vaginal human isolates) from the acidophilus group, stably presented in the human microbiome, was not observed by the agar diffusion assay (unpublished data). This may have an impact on further clinical application, based on the fact that all strains tolerate each other well, and thus are appropriate for multibacterial probiotic LAB formulas. The anti-*Staphylococcus* activity detected in milk samples and combined effects between two strains, *L. salivarius* 1 and *L. plantarum* L6, have a practical application for food safety. The detected antibacterial activity of postmetabolites (10% *v*/*v* added to BHI broth) against (a) *E. coli* WDCM 00013, (b) *S. aureus* NCTC 6571, (c) *S. mutans* DSMZ 20523, and (d) *P. aeruginosa* WDCM VT00114 simulate a possible effect *in vivo*. *L. salivarius* activity toward various planktonic and biofilm-forming *S. aureus* has been proven [43]. In addition, viable and heat-killed cells of *L. salivarius* AP-32 were deemed active toward distinct oral pathogens, including *S. mutans* [44].

A wide spectrum of activity has been determined for *L. plantarum* in various studies, such as the activity of CFS toward *P. aeruginosa* and methicillin-resistant *S. aureus* (MRSA) [45], *E. coli* [46], and *S. mutans* [47]. Such activity of *L. plantarum* DY7 (out of seven tested strains) toward *E. coli*, *S. aureus*, and *S. enterica* serovar Typhimurium has also been shown [46]. In addition, the inhibition of *E. coli* growth by CFS was reported at different time points. The highest effect was reported when the CFS was added at the start point (0 h), similar to our experiments. The possible acidification likely resulted in pathogen growth inhibition and the prolonged lag phase observed (Figure 2). On the other hand, addition of CFS from *L. plantarum* DY7 to the late logarithmic phase (after 6 h) did not affect the growth of *E. coli.* The major detected substances produced were lactic acid, acetic acid, propionic acid, and caprylic acid [46].

### 3.2. In Vitro Assessment of Antifungal Activity of LAB Strains

**Figure 3:**
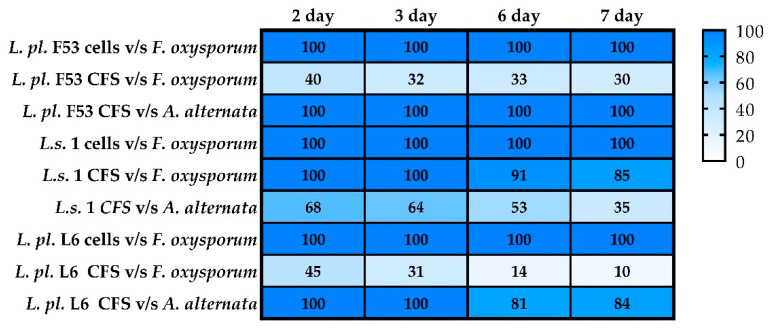
In vitro evaluation of the antifungal activity of LAB cells (by mono- or two-layer methods) and 10% *v*/*v* CFS of *Lactobacillus* cultures in MRS broth, expressed as % inhibition against the control (tested fungal strains: *F. oxysporum* spp.; *Alternaria* spp.). All samples were tested in triplicate and subjected to statistical analyses using one-way ANOVA followed by Tukey’s post hoc test; *p* < 0.001.

A promising antifungal potential of four LAB strains against *A. fumigatus* 3-2, *F. oxysporum* NBIMCC 124, and *A. alternata* NBIMCC 110 was shown. An adapted protocol using PDA and MRS agar allowed the estimation of antifungal activity in vitro using cell-free supernatants and postbiotics (living cultures with CFS) inoculated (10% *v*/*v*) in PDA plates. An additional test using the overlay method against *Fusarium oxysporum* was carried out, assessing the activity of the dynamically produced metabolites (Figure 3). The results of fungal growth inhibition (as % of the control) are summarized in Figure 3.

**Figure 4:**
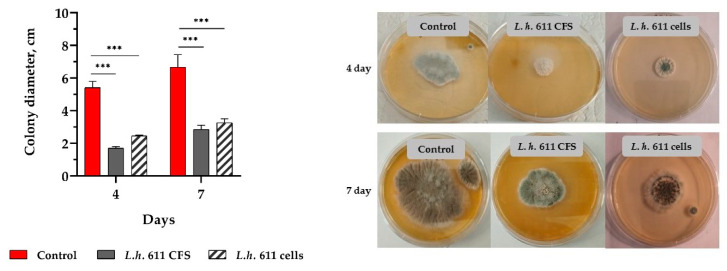
In vitro inhibitory activity against *A. fumigatus* strain 3-2 of CFS and exponential cultures of strain *L.h.* 611 in MRS broth inoculated in PDA agar plates (10% *v*/*v*). The monolayer method with PDA and a control containing 10% *v*/*v* MRS broth (pH 4.5) were used. The data from triplicate tests (mean ± SD) were subjected to statistical analyses, and the differences between samples and control were assessed using one-way ANOVA followed by Tukey’s post hoc test (*** *p* < 0.001).

Differential inhibitory effects, depending on the LAB strains, the forms (postbiotics, living cells, and/or CFS), and the target fungus, were found in a triplicate assay. The cell cultures of tested LAB strains inoculated in PDA (10% *v*/*v*) completely inhibited *F. oxysporum* growth (Figure 3). At the same time, *L. helveticus 611* inhibited *A. fumigatus* colony growth (Figure 4). Stronger inhibition was demonstrated for CFS compared to cell cultures of the tested strains (Figure 4). A varying degree of inhibition of tested LAB CFS was observed. Generally, CFS showed lower inhibition against *F. oxysporum* than cell cultures. The highest inhibition of *A. alternata* growth exhibited CFS of *L. plantarum.* F53, followed by *L. plantarum* L6 (Figure 3). A strain of this species, isolated from camel milk, inhibited the growth of *A. alternata* by about 66.7% [48].

Fungi, such as *Fusarium*, *Aspergillus*, *Candida* spp., etc., are causative agents of opportunistic fungal infections in immunocompromised individuals and are gaining increased drug resistance. *Aspergillus* spp. (*A. flavus*, *A. fumigatus*, *A. nidulans*, etc.) cause allergies (aspergillosis) in more than 45% of immunocompromised patients [49], and *Fusarium* spp. have been linked to systemic or local invasive infections [50].

The LAB and their postbiotics have been reported to possess a wide spectrum of antifungal activity [17,51]. Variants in vitro tests showed strain-specific antifungal activity (Figure 3 and Figure 4). Moreover, living cells and produced metabolites in dynamics during the cultivation in MRS agar do not allow growth of spores of *F. oxysporum*, *A. alternata*, and *A. fumigatus*. Thus, the pre-selected lactobacilli are suitable for development as biopreservative adjuncts to starter cultures in fermented products. LAB with the most pronounced antifungal activity have the potential for application in food technology [48].

The non-purified CFS of LAB showed varying degrees of inhibition, which is likely due to the decreased concentration or stability of antifungal metabolites over time (Figure 4). However, metabiotics of *L. plantarum* strains (*L.pl.* F53 and L6) possessed higher activity toward *A. alternata*, suggesting that specific produced molecules in the spent cultures play a significant role against this mold. *L. plantarum* is well-known for its antifungal activity being proven against various fungi [52]. The species is genotypically heterogeneous, and phenotypic variation exists among the antifungal metabolites produced [53]. A strain-specific antifungal spectrum of 88 *L. plantarum* strains against several fungi, such as *A. niger*, *P. chrysogenum*, *A. flavus*, *P. roqueforti*, *P. expansum*, *F. culmorum*, and *Cladosporium* spp., was shown [54]. *L. salviarius* 1 and *L. helveticus* 611 also demonstrated broad antifungal activity. In a study by Hu et al. (2017), *L. salivarius* CFS inhibited conidial germination and mycelial growth of *Fusarium solani by* possible proteinaceous compounds produced [28].

Recent data have revealed diverse LAB metabolites that alter the physiology of molds by inducing oxidative stress and ATP depletion, leading to cytotoxicity or growth inhibition. Other metabolites compromise the structural integrity of the fungal cell, leading to alterations in cell morphology, membrane permeability, and death, while biosurfactants prevent adhesion to mucosal surfaces [55].

### 3.3. Radical Scavenging Activity of Metabiotics from LAB Cultures

**Figure 5:**
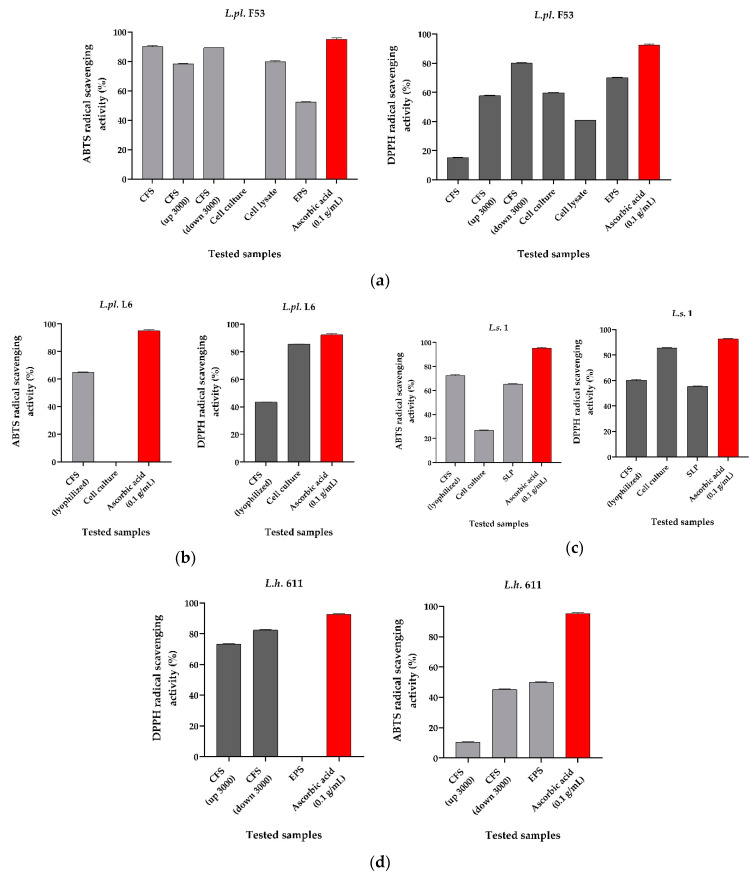
The DPPH and ABTS radical scavenging activity of metabiotics from tested LAB strains: (**a**) *L. plantarum* F53; (**b**) *L. plantarum* L6; (**c**) *L. salivarius* 1; (**d**) *L. helveticus* 611. Ascorbic acid (0.1 g/mL) was used as a positive control. All experiments were performed in triplicate to ensure reproducibility and accuracy, and the average values were used for analysis. The data are presented as mean ± SD for each group (*n* = 3); *p*-values were calculated using one-way ANOVA followed by Tukey’s post hoc test in comparison to ascorbic acid (*p* < 0.001). Legend: red bars—positive control used (ascorbic acid) in the both variants of assay; gray bars—samples tested by ABTS method; dark grey bars—samples tested by DPPH method.

The DPPH- and ABTS-radical scavenging activity of four live *Lactobacillus* cells (in suspension with PBS, pH 6.5) and CFS were assessed in vitro (Figure 5). A strain-specificity with a visible difference between the ABTS and DPPH results for tested CFS and cells of exponential cultures in MRS broth (pH 6.5) was noted. All four lactobacilli showed promising activity, comparable to that of the positive control, ascorbic acid (0.1 g/mL). In both variants, high activity of *L. helveticus* 611 and *L. plantarum* L6 was measured (Figure 5).

Probiotic LAB and their active metabolites produced during fermentation are receiving increasing attention as antioxidants with beneficial effects on human health. Their involvement in the prevention of negative effects of cellular/oxidative stress is relatively little studied. The antioxidant properties of cell-free supernatants may be due to various secreted molecules. The extracellular pyruvate produced by *Lactococcus lactis* ATCC 19435 can scavenge H_2_O_2_ generated by NADH oxidase [56].

The CFS from *L. plantarum* F53 culture (from a vaginal sample) and from *L. plantarum* L6 (isolated from “katak”, a fermented milk product) displayed a moderate (43.38 ± 0.27%) radical scavenging activity by the DPPH method and a high (64.95 ± 0.34% for L6 to 90 ± 1% for *L.pl*. F53) activity by ABTS technology (La Crosse, WI, USA), respectively. Highly promising are the results for *L. helveticus* 611 CFS with 10.5% (for concentrated upper phase of CFS using 3000 Da cut-off membrane) and 52 ± 03%, respectively, for the ABTS method. Experimental results of intact cells of *L. salivarius* 1 (84.58 ± 0.34%), *L. plantarum* L6 (84.58 ± 0.34%), *L. plantarum* F53 and those obtained for cell-free spent cultures showed stronger DPPH activity. High scavenging of DPPH free radicals from CFS from *L. plantarum* F53 and *L.h.* 611 (Figure 5d), in samples of CFS concentrated by an Amicon membrane with a 3000 Da cut-off (73.2 ± 2.73%), may be attributed to the hydrogen-donating ability of tested postbiotics in vivo. The calculated results, as well as the CFS of *L. helveticus* 611 (sample labeled ‘down’), were diluted 10 times prior to incubation with DPPH/ABTS reagents. According to Sánchez-Moreno (2002), the structural configuration of the DPPH radical prevents high molecular weight antioxidants from interacting with it [57]. The higher activity we obtained with the DPPH method could be explained by the fact that the overall antioxidant activity of the cell suspension can be increased by disrupting the tertiary structure of some cellular proteins and thus promoting accessibility to amino acid residues that can inactivate free radicals [58].

Various studies on lactobacilli present LAB ability to scavenge free radicals in vitro [59] and in vivo [60]. Published data demonstrate that these bacteria can induce positive effects in the microbiome, preventing many diseases associated with oxidative stress [61].

ABTS radical scavenging activity of lyophilized CFS from exponential *L. salivarius* 1 culture is also promising (−72.37 ± 0.59%; Figure 5c). The lyophilized form is an analogue of a ready-to-use probiotic that contains living cells and concentrated metabiotics. In this study, the antioxidant capacity of *L. plantarum* F53 (sample ‘CFS up’) of −78.2 ± 0.34% against ABTS radical is also estimated as an important index for this strain.

The ABTS/DPPH scavenging abilities of the isolated crude EPS from CFS (F53) (−52.40 ± 0.11% and 70.26 ± 0.14%. for *L.s.* 1 lysates and *L.s.* 1 surface layer protein (SLP), respectively) are also promising as possible in vivo effects (Figure 5c).

According to Tang et al. (2017), the activity of cell-free spent cultures may be due to extracellular production of exopolysaccharide with a high content of sulfhydryl groups (–SH) [62]. *L. lactis* subs. *lactis* 12 and *Bifidobacterium animalis* RH have the ability to produce extracellular polysaccharides and to trap hydroxyl (HO^•^) and superoxide (O^2•−^) radicals. Strong antioxidant effect and scavenging ability for HO^•^ and O^2•−^ has been found [62].

Our study confirms the published data by Zhang et al. (2013) on supernatants from LAB, with antioxidant activity [63]. Eldawi et al. (2012) determined very high values of DPPH activity for *L. plantarum* for both intact cells (51–86.5%) and supernatants (58–89%) [64]. In other studies of cell lysates and intact cells, a wide variation between strains, from 11.3% to 53.2%, was reported [65]. Similar to our results, higher activity was shown for intact cells [65]. The highest antioxidant activity for nine tested isolates was found for *Lactobacillus delbrueckii* subsp. *bulgaricus* F17 (22.57%) according to DPPH in cell suspension assays [66].

### 3.4. EPR Analysis of Ready-to-Use Probiotics from Two Selected Strains

**Figure 6:**
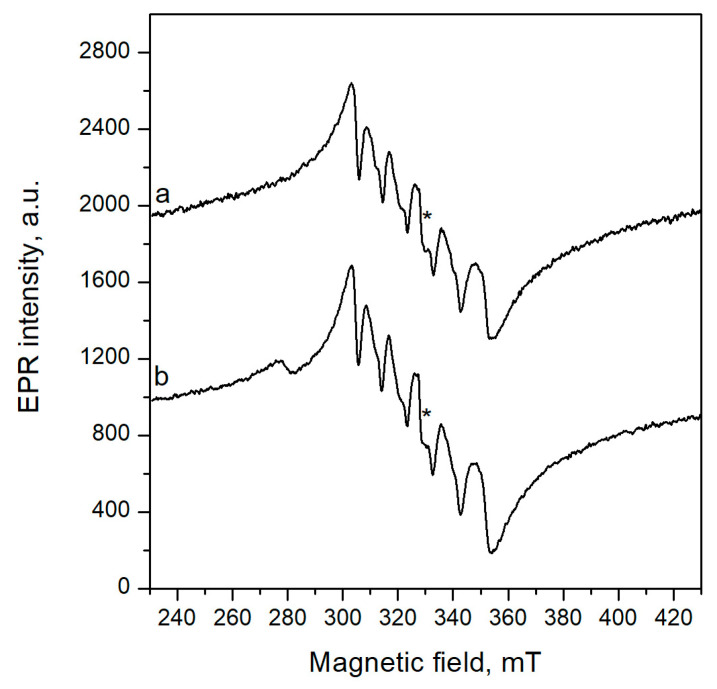
EPR spectra of lyophilized samples simulating real probiotic powder from (a) *L. salivarius* 1 and (b) *L. plantarum* L6. Legend: The asterisk denotes the signal with a g factor of 2.0012 ± 0.0005.

Additional characterization of possible mechanisms of the antioxidant potential of selected active lactobacilli was made by EPR spectroscopic analysis. The EPR spectra of lyophilized form of *L. salivarius* 1 and *L. plantarum* L6 samples at room temperature are shown in Figure 6. They contain six lines with g values of 2.1543, 2.1068, 2.0562, 2.0012, 1.9316, and 1.8658. The constant of hyperfine splitting increases at a higher magnetic field. The average hyperfine coupling constant “A” is approximately 9.1 mT. This indicates the presence of the paramagnetic Mn^2+^ ions in the samples. Mn^2+^ has a half-filled *d* shell (3d^5^) with angular momentum L = 0 and spin S = 5/2. The hyperfine lines are due to the interaction between the unpaired electron with the ^55^Mn nucleus with nuclear spin I = 5/2. The hyperfine lines correspond only to the M_s_ = |−1/2〉 to Ms = 〈+1/2| transition since in powders, as in our case, all other transitions are broadened due to the zero-field splitting and contribute to the total background upon which the signal is superimposed. Several shoulders between the major six hyperfine lines can be observed. These shoulders originate from the presence of additional Mn (II) species or from forbidden transitions (ΔM_S_ = ±l and ΔM_I_ = ±l), common in Mn (II) spectra. On the other hand, in both samples, a tight signal with a g factor of 2.0012 ± 0.0005 (denoted with an asterisk at the spectra in Figure 6) was recorded. According to the literature, the signal with this g factor of Lande is naturally present in mushroom samples [67]. On the other hand, the EPR peak at g ≈ 2.001–2.004 can be attributed to oxygen vacancies.

In addition, in the spectrum of L6, a weak line at g = 2.3474 was recorded. The g-value of ≈2.3 is characteristic of ferromagnetic Ni^0^ particles [68]. Another assumption is that it could be due to Ni^+^ ions [69].

Lactobacilli may possess the ability to interact with metal ions with a role in oxidative stress response, which can be studied using Electron Paramagnetic Resonance spectroscopy (EPR). EPR spectroscopy plays an important role in understanding organic and inorganic radicals, transition metal complexes, and some biomolecules. The EPR method is well suited for determining the radical-scavenging ability of substances. It is characterized by high sensitivity and selectivity. Such an innovative experimental approach is appropriate to observe labeled species in situ, either biologically or in a chemical reaction. Therefore, the two LAB strains were prepared in lyophilized form to simulate a real probiotic product and their potential for metal/radical scavenging efficiency in vivo. Some lactobacilli are known for their ability to interact with metal ions and their role in oxidative stress response. Probiotic *L. paracasei* ATCC 55544 accumulate manganese, which may act as a free radical scavenger to maintain viability, during long-term storage at room temperature [70]. Preliminary EPR measurements in our tests revealed paramagnetic particles (substances) and free radicals in the sample. *Lactobacillus* species often accumulate manganese as a protective mechanism against oxidative stress [70]. The detection of Mn^2+^ ions in the samples is significant, as it suggests that the *Lactobacillus* bacteria in these samples may utilize Mn^2+^ for their antioxidant properties [71]. Mn^2+^ acts as a scavenger of reactive oxygen species (ROS), protecting LAB cells from oxidative damage [71]. The presence of Mn^2+^ ions in the EPR spectra aligns with the known protective roles of manganese in *Lactobacillus* cells against oxidative stress. Moreover, manganese plays an important role in proper functioning, gene and homeostasis regulation, electron and biological substance transport, signal transduction, the stability of the metalloproteins and metalloenzymes, biomolecule synthesis, and oxidative stress [72,73].

Confirming the obtained EPR data, a bioinformatic analysis (using the Prokka tool integrated into Galaxy EU) on the whole genome sequencing of the tested strain *L. plantarum* F53 showed the presence of the manganese transport system ATP-binding protein MntB. The g-factor signal around 2.0012 suggests the presence of radicals, which could be linked to oxidative stress responses in *Lactobacillus*. This may indicate active metabolic processes or microbial responses to stress conditions. However, additional analyses are needed to find more information. To our knowledge, these are the first data on the estimation of LAB by EPR analysis. The initial results obtained give us reason to believe that EPR spectroscopy can estimate the radical-scavenging capacity of the examined samples. This requires further studies of mycelial solutions with different concentrations and the use of so-called “spin-catching”, which will be the subject of future studies [70].

### 3.5. FPLC Chromatography Analyses

**Figure 7:**
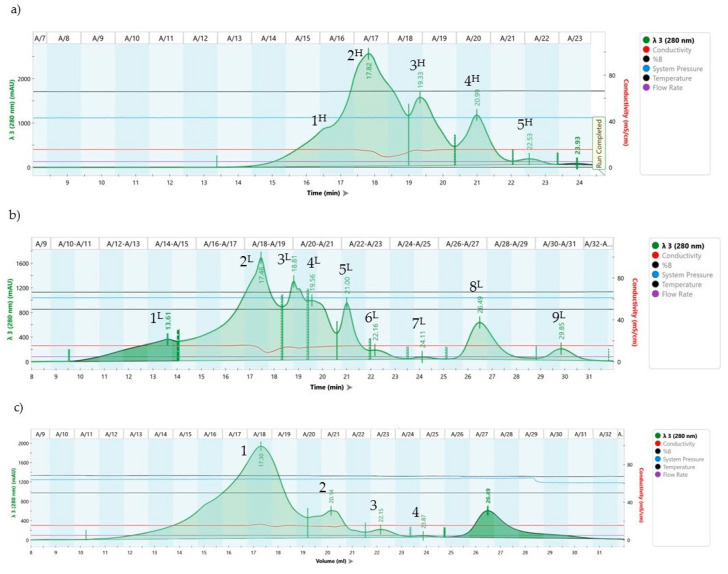
Results from FPLC chromatographic analysis, presenting SEC profiles of filtered CFS of both tested *Lactobacillus* cultures in MRS broth (pH 6.5), on a Superdex Peptide 10/300 GL columns (SEC 70kDA and SEC 650 kDa CFS) as follows: (**a**) CFS of *L. helveticus* 611 at SEC by 650 kDA; (**b**) CFS of *L. helveticus* 611 by SEC 70 kDA, and (**c**) CFS of *L. plantarum* F53 by SEC 70 kDA. Legend: The fractions are numbered in the order of their manual collection. CFS peaks of *L.h* 611 collected from SEC 70 kDA are designated L (as low) or H (as high) from SEC 650kDA respectively.

Our promising results with CFS with radical scavenging and a broad spectrum of activity are a base to learn more about the nature of active molecules/postmetabolites produced during fermentation. To this aim, a method of FPLC chromatography analysis was applied using SEC 70 and SEC 650. A chromatographic analysis of the cell-free spent cultures of two active strains, *L. plantarum* F53 and *L. helveticus* 611, was performed. Initially, the CFS from both strains were subjected to ultrafiltration on a Pellicon^®^ XL Cassette with Durapore^®^ Membrane Millipore system to remove unwanted impurities before application to the FPLC column system. According to the optimized protocol, with an experimentally determined optimal pH of 7.5, an elution buffer with 0.01 M NaCl was applied, respecting the optimal parameters for columns SEC 70 and SEC 650. The separation of CFS from late exponential phase cultures of the CFS *L.h*. 611 and CFS *L.pl.* F53 is presented in Figure 7a–c.

Late exponential phase CFS of *L. salivarius* 1 and *L. helveticus* 611 strains were fractionated using two types of columns, SEC 70 and SEC 650.

The initial calibration of the column with protein markers/standards was in a wide range to allow the determination of low and high molecular mass fractions. Sample CFS, *L.h*. 611, was evaluated on both columns and showed better separation of SEC 70 kDa. A difference in the chromatographic profile of the two strains is well illustrated with presented profiles from low molecular weight gel filtration column analysis (Figure 7a,b). All fractions from CFS of *L. helveticus* 611, marked with numbers and a superscript suffix ^H^ for Colum SEC 650 and with ^L^ for Sec 70, respectively, were collected manually. The nine peaks in the range from ~70,000 Da to ~500 Da were stored at −20 °C prior to following MTT analysis. On the chromatogram for *L.pl.* F53 CFS (Figure 7c), four peaks in the range of approximately 2 kDa to 0.05 kDa are clearly detected, with the high molecular weight column SEC 650.

Gel-filtration analysis was performed to fractionate the cell-free spent cultures, CFS *L.pl.* F53 and CFS *L.h.* 611, with expressed antioxidant and antimicrobial activity. The choice of FPLC–size exclusion chromatography (SEC) was made as it is a simple and reliable technique, for initial separation of molecular components according to their size. Regardless of limitations in flow and sample volume, the technique allows removal of aggregates of the target substance, buffer exchange, and salt removal. This was our approach to separate produced metabolites based on the difference in molecular mass and to assess their beneficial biological potential. The differences in the FPLC profiles of both strains, *L. helveticus* 611 and *L. salivarius* 1, may correspond to the different metabolites produced during the fermentation. Therefore, the collected fractions (Figure 7a–c) were used in the subsequent MTT assay. Additional optimization of combinations with other chromatographic methods, however, is needed and would be useful in further characterization.

### 3.6. Inhibitory Effects of Postbiotics of LAB Cultures on the Growth of K562 Cell Lines Using the MTT Assay

The K562 cell line is a typical model for chronic myeloid leukemia. The line has been convenient in studying the effects of various therapeutic agents due to its stable phenotype and karyotype that are preserved after long cultivation periods [74]. Chronic myeloid leukemia is a hematopoietic stem cell disorder affecting both blood and bone marrow, accounting for approximately 15% of newly diagnosed adult leukemia cases [75]. Different LAB strains have already entered the probiotic market, and novel strains with more functional characteristics are needed due to the growing demand for new natural anti-cancer molecules.

**Figure 8:**
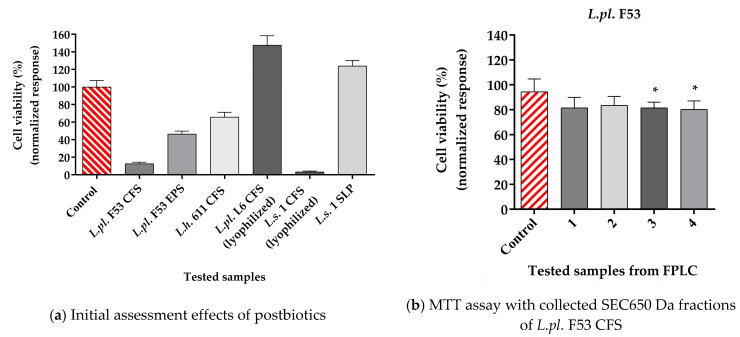

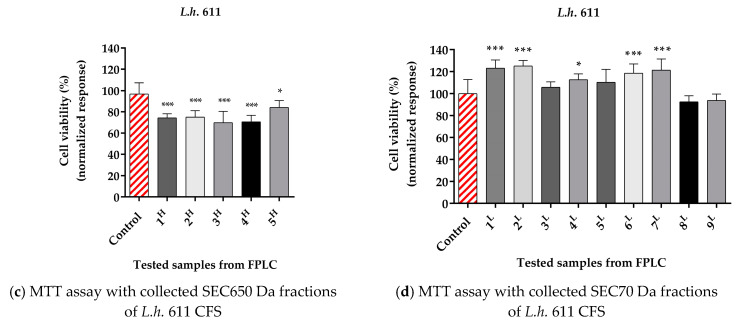
MTT assay to establish inhibitory effects on the K562 cells of (**a**) postbiotics; (**b**) SEC650 Da fractions of *L.pl.* F53 CFS; (**c**) *L.h.* 611 CFS; and (**d**) SEC70 Da fractions of *L.h.* 611 CFS, collected from FPLC analysis. The postbiotics and fractions of CFS were supplemented (4% *v*/*v*) to the cells in RPMI. The controls used are K562 cells in RPMI media inoculated with 4% *v*/*v* MRS, pH 6.5 (**a**) and 4% *v*/*v* elution buffer (**b**–**d**)—the red bars The data from eight replicates (mean ± SD) were subjected to statistical analyses, and the differences between the samples and control were assessed using one-way ANOVA followed by Tukey’s post hoc test (* *p* < 0.05; *** *p* < 0.001). All results from (**a**) show *p* < 0.001.

The cell growth inhibition capacity of the candidate-probiotic lactobacilli was performed on exponentially grown K562 cells using the MTT assay. Initial screening was performed with different postbiotics obtained during fermentation (CFS *L.pl.* F53 and *L.h.* 611), SLP (surface layer protein) from *L. salivarius 1*, and lyophilized forms (probiotics *L.pl.* 6 and *L.s.* 1) (Figure 8a). The obtained SEC-FPLC fractions of spent cultures of both *Lactobacillus* strains *L.pl.* L6 and *L.s.*1 were estimated (Figure 8b–d). The inhibition ratio (%) was variable, ranging from strong inhibition to stimulation (Figure 8a–d).

**Figure 9:**
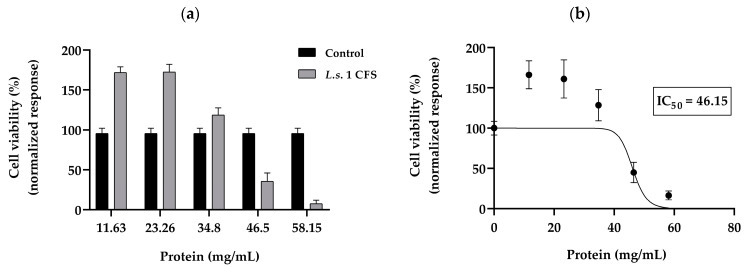
MTT assay to determine (**a**) effect of active CFS of *L. salivarius* 1 strain on the cell viability of the K562 cell line and (**b**) determination of the IC50 value. The data from (**a**) are presented as mean ± SD for each group (*n* = 8); *p*-values were calculated using one-way ANOVA followed by Tukey’s post hoc test in comparison to control (K562 cell in RPMI media inoculated with 4% *v*/*v* MRS, pH 6.5). All results from (**a**) show *p* < 0.001.

Our study demonstrates that the tested strains, particularly *L. pl.* F53 and *L.s.*1 significantly inhibit the growth of K562, with the strongest effect for CFS (12.5% and 4.5% cell viability for the respective strains) (Figure 8a). Lower inhibition was found for *L.pl.* F53 exopolysaccharide tested and CFS from *L.h.* 611, while no effect was observed for SLP of *L.s*.1 strain (Figure 9a).

A difference in protein content (determined by the Bradford method) between all tested samples should be pointed out. It is difficult to connect the stimulation of cancer cells only with protein concentration. We assume that active metabolites, produced during LAB fermentation, similar to activity of natural plant-origin drugs, may influence metabolic pathways of cancer cells. Thus, using a multitude of biological mechanisms, they may modulate or promote proliferation or apoptosis in cancer cells.

The CFS fractions obtained by SEC-FPLC chromatography analysis revealed viability equal to approximately 80% of treated K562 cells. The summary of results indicates that the fractions contain bioactive compounds with varying effects on cell viability, from inhibition to stimulation (Figure 8b–d). The cell viability of K562 cells following treatment with high molecular weight fractions from *L.pl.* F53 and *L.h.* 611 was in the range of 70–80%, while exposure to two of the low molecular fractions led to 92–93% growth, respectively (Figure 8b–d). CFS fractions > 100 kDa, produced from *Limosilactobacillus reuteri*, were reported as inductors of apoptosis in the colon cancer cell line HT29, possibly due to secretory macromolecules such as a proteins, polysaccharides, and nucleic acid [76]. Kim et al. (2008) demonstrated that protein components from *Lacticaseibacillus casei* exhibited significant anti-cancer activity while showing low cytotoxicity to normal cells [77].

In our study, K562 cells were treated with different protein concentrations of *L.s.* 1 CFS (between 11.6 and 58.1 mg/mL protein) for 72 h, and the MTT assay was carried out to determine the effects of postbiotics on viability (Figure 9a). At 46.15 mg/mL of the CFS *L.s.* 1 treatment, 50% cell growth inhibition was achieved (IC_50_). Concentrations of *L.s.* 1 at 58 mg/mL and above led to 80–90% cell growth inhibition. In contrast, concentrations one-, two-, and four-fold lower than IC_50_ of *L.s*. 1 (34.8 mg/mL, 23.26 mg/mL, 11.63 mg/mL, respectively), exerted a stimulatory effect over the treated K562 cells that reached 166% growth compared to the respective control at 11.63 mg/mL. The IC_50_ value was 46.15 mg/mL (Figure 9b).

The role of LABs’ CFS and other cell components on the growth of K562 and other cancer cells has been evaluated in vitro by other authors. Chuah et al. (2019) show the anti-proliferative/cytotoxic and strain-specific effects of postmetabolites produced by six *L. plantarum* strains for normal human primary cells, and breast, colorectal, cervical, liver, and leukemia K562 cell lines [78]. Promising is the activity of tested postbiotics (heat-killed cells, bacterial cell wall extract, and genomic DNA) of eight strains belonging to the species *L. rhamnosus*, *L. paracasei* ssp. *paracasei*, *Loigolactobacillus coryniformis* subsp. *torquens*, which exerted an antiproliferative effect on the K562 cell line [79]. The available literature suggests that the imbalance of the gut microbiome could contribute significantly to an increased risk of relapse for people living with chronic myeloid leukemia (CML) and acute lymphoblastic leukemia (ALL), supporting the idea to study effects on the K562 cell line, a proven model for CML [80]. Further research is necessary to investigate the link between immunomodulatory capacity of the healthy gut microflora and hemotherapy success in patients with hematological malignancies. The obtained data are far from real pro-/postbiotics with direct pharmaceutical activity in case of such deadly cancer. However, when the host gut microbiota predisposes the patient to relapse given its potent immunomodulatory capacity, targeted mechanisms to reverse dysbiosis as a factor of illness are needed.

## 4. Conclusions

Our conception to study such a panel of in vitro activity is to estimate the probiotic potential, based on production of active postbiotics/metabiotics. Living cells with such metabolic activity may contribute to gut eubiosis, in vivo, in case of oral administration. Postbiotics produced in situ, during growth and fermentation in the colon, may contribute to reduce inflammation (provoked by pathogens) and the negative consequences of oxidative stress.

In conclusion, the pre-selected *L. plantarum L6* and *F53*, *L. salivarius 1*, and *L. helveticus 611* strains were characterized as promising candidate probiotics. They possessed a strain-specific broad spectrum of antibacterial and antifungal activity, supporting natural barrier mechanisms against invading pathogens. The proven high radical scavenging activity in vitro and specific inhibitory effect on the growth of K562 chronic myeloid leukemia cells make them suitable for the development of target probiotics/postbiotics, with specific beneficial effects.

We seek to apply these activities further in the design of target pro-/postbiotics that are helpful to solve a specific health problem. Further investigations on the nature of the produced postmetabolites and *Lactobacillus* safety must be completed before they can be considered as scientifically proven probiotic strains with possible health benefits.

## Data Availability

Data are contained within the article.
